# A Gut Feeling: Metastatic Colorectal Adenocarcinoma With Dirty Necrosis in a Young Adult Without Risk Factors

**DOI:** 10.7759/cureus.105968

**Published:** 2026-03-27

**Authors:** Kian Memari, Emily Reinoso, Anastasiya Sizova, Lissette P Lazo, Shane Williams, Peter Cohen

**Affiliations:** 1 Family Medicine, Palmetto General Hospital, Miami, USA; 2 Medicine, Nova Southeastern University Dr. Kiran C. Patel College of Osteopathic Medicine, Fort Lauderdale, USA; 3 Family Medicine, Nova Southeastern University Dr. Kiran C. Patel College of Osteopathic Medicine, Fort Lauderdale, USA

**Keywords:** dirty necrosis, early-onset colorectal cancer, iron deficiency anemia, liver metastasis, rectal adenocarcinoma, screening guidelines

## Abstract

Colorectal cancer (CRC) is traditionally considered a disease of older adults with established risk factors such as family history, inflammatory bowel disease, or hereditary syndromes. However, CRC is increasingly being diagnosed in individuals younger than 50 years of age, a phenomenon referred to as early-onset CRC (EOCRC). These patients frequently present with advanced disease and often lack classic predisposing factors, posing diagnostic and clinical challenges.

We report the case of a 38-year-old male patient with no personal or family history of malignancy who presented with approximately four weeks of abdominal pain, tenesmus, and hematochezia. Imaging revealed circumferential rectal wall thickening with a large hepatic mass and lymphadenopathy. Colonoscopy demonstrated a nearly circumferential friable rectal mass extending into the anal canal. Histopathologic evaluation of the liver lesion revealed metastatic adenocarcinoma characterized by extensive dirty necrosis, strongly favoring a colorectal primary. Serum carcinoembryonic antigen (CEA) was markedly elevated. Based on imaging and histopathologic findings, the disease was staged as stage IV CRC with hepatic metastasis.

This case highlights the aggressive nature of EOCRC, the diagnostic importance of pathologic features such as dirty necrosis in determining tumor origin, and the need to reconsider diagnostic thresholds when younger adults present with gastrointestinal symptoms.

## Introduction

Colorectal cancer (CRC) is the third most commonly diagnosed malignancy worldwide and the second leading cause of cancer-related mortality [1]. Historically, CRC predominantly affected individuals older than 50 years of age, which shaped traditional screening recommendations. Common clinical manifestations include rectal bleeding, iron deficiency anemia, abdominal pain, changes in bowel habits, weight loss, and tenesmus [2].

Over the past several decades, however, epidemiologic studies have demonstrated a concerning rise in early-onset CRC (EOCRC), defined as CRC diagnosed before the age of 50. EOCRC currently accounts for approximately 10% to 12% of newly diagnosed colorectal cancers, and its incidence continues to increase globally [2].

Compared with late-onset disease, EOCRC frequently demonstrates distinct clinicopathologic characteristics, including a higher prevalence of rectal tumors, a more advanced stage at diagnosis, and aggressive histologic subtypes such as mucinous or signet-ring differentiation [3]. Many affected patients lack identifiable hereditary syndromes or classical risk factors, which may contribute to delayed diagnosis and treatment [4].

Histopathologic evaluation can provide critical diagnostic clues when metastatic lesions are identified. One such feature is “dirty necrosis,” characterized by intraluminal necrotic debris consisting of apoptotic tumor cells, inflammatory cells, and nuclear fragments within malignant glandular structures. Dirty necrosis is considered a characteristic histologic hallmark of colorectal adenocarcinoma and can help distinguish metastatic colorectal lesions from primary tumors arising in other organs, such as the liver or pancreas [5].

We present a case of metastatic rectal adenocarcinoma in a 38-year-old male patient without known hereditary risk factors, in which the presence of extensive dirty necrosis in metastatic liver tissue played a key role in identifying the colorectal origin of the malignancy. This case underscores the importance of maintaining clinical vigilance when evaluating gastrointestinal symptoms in younger adults.

## Case presentation

A 38-year-old male patient with no significant past medical history presented to the emergency department with approximately four weeks of progressively worsening generalized abdominal pain, tenesmus, and hematochezia with clot formation. The hematochezia had been present for approximately three weeks prior to presentation and had increased in frequency during the week before admission. The patient denied melena, nausea, vomiting, diarrhea, unintentional weight loss, or night sweats. He reported a long-standing history of constipation since childhood, but had never undergone colonoscopic evaluation. There was no personal or family history of colorectal malignancy, inflammatory bowel disease, or hereditary cancer syndromes. Social history revealed a diet predominantly composed of red and processed meats with frequent fast-food consumption and minimal dietary fiber. The patient had never established care with a primary care physician.

On presentation, vital signs were notable for low-grade fever and hypertensive urgency, requiring treatment with an intravenous nicardipine infusion. Physical examination demonstrated mild diffuse abdominal tenderness without rebound or guarding. Digital rectal examination was limited due to discomfort but revealed gross blood. Laboratory studies demonstrated moderate normocytic anemia with iron deficiency, including low serum iron and ferritin levels. The patient received intravenous iron sucrose (Venofer®) 400 mg for iron-deficiency anemia. Liver function tests and lipase were within normal limits. Serum carcinoembryonic antigen (CEA) was markedly elevated at 382 ng/mL (Table 1).

**Table 1:** Laboratory values U/L: Units per liter, TIBC: Total iron binding capacity, AST: Aspartate aminotransferase, ALT: Alanine aminotransferase, HDL: High-density lipoprotein, CEA: Carcinoembryonic antigen

Test (Unit)	Observed Value	Normal Range
Hemoglobin (gm/dL)	11.8 gm/dL	14.0-18.0 gm/dL
Iron (mcg/dL)	47 mcg/dL	49.0-181.0 mcg/dL
TIBC (mcg/dL)	418 mcg/dL	261-462 mcg/dL
Saturation (%)	11%	20-55%
Transferrin (mg/dL)	336.7 mg/dL	206.0-381.0 mg/dL
Ferritin (ng/mL)	11.8 ng/mL	17.9-464.0 ng/mL
AST (U/L)	36 U/L	17-59 U/L
ALT (U/L)	12 U/L	21-72 U/L
Alkaline phosphatase (U/L)	105 U/L	38-126 U/L
Total cholesterol (mg/dL)	206 mg/dL	<200 mg/dL
HDL (mg/dL)	26 mg/dL	40-59 mg/dL
CEA (ng/mL)	382 ng/mL	0.0-3.0 ng/mL

**Figure 1:**
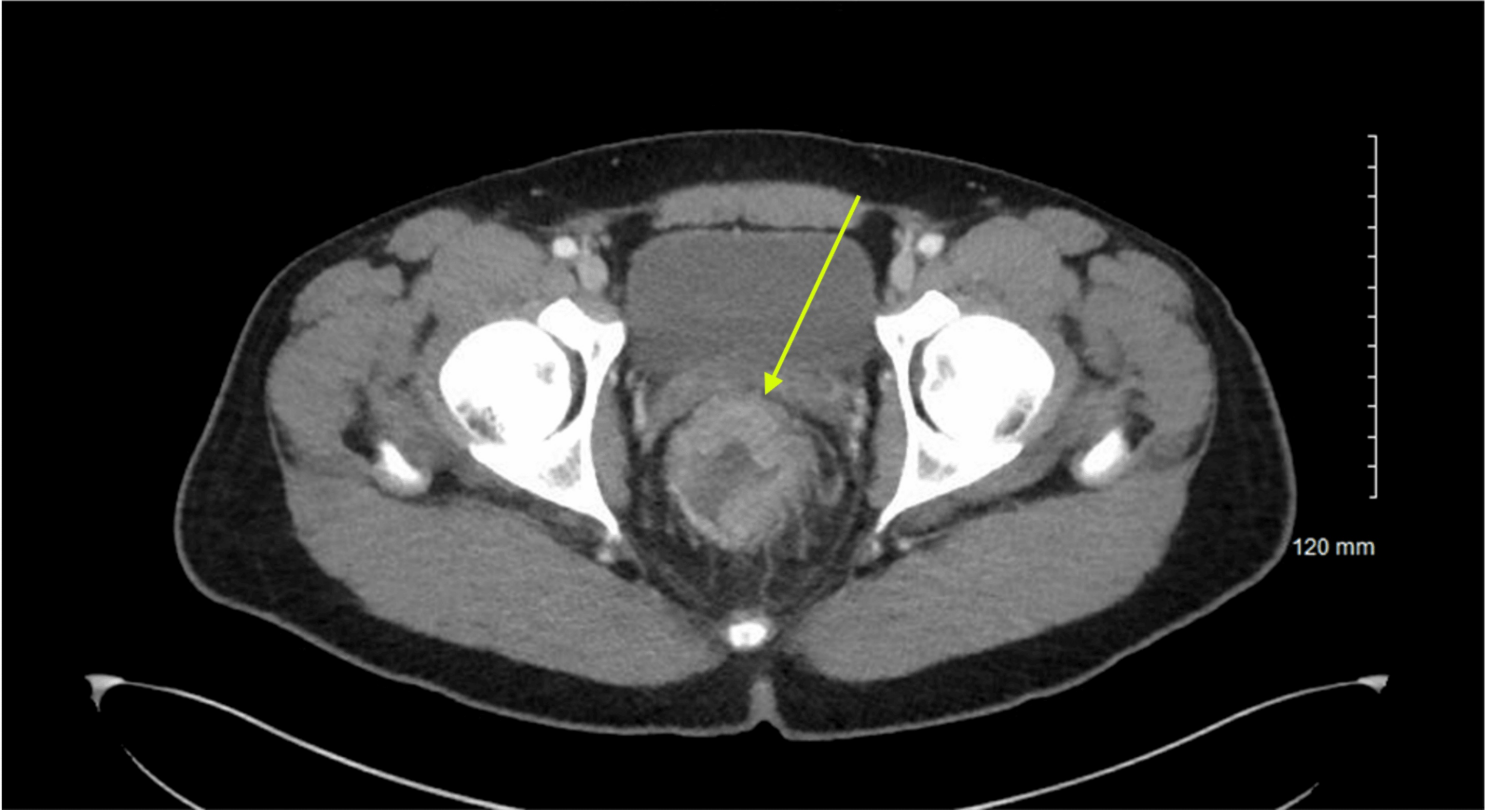
Rectal malignancy The yellow arrow indicates circumferential rectal wall thickening up to 2.3 cm, suspicious for rectal malignancy.

**Figure 2:**
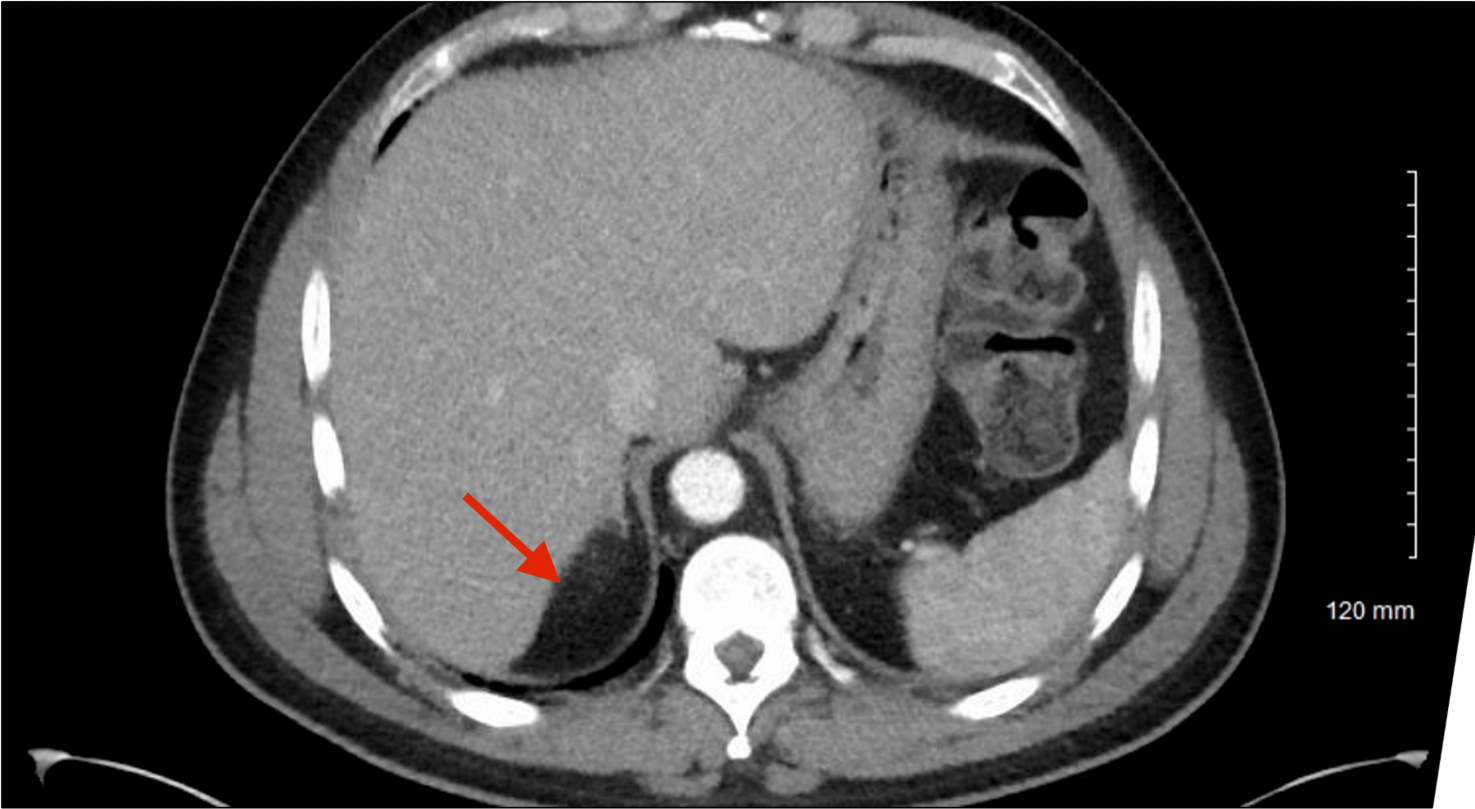
Liver lesion The red arrow indicates a hypodense lesion measuring 8.7 x 10 x 7.5 cm in the posterior right lobe of the liver.

Computed tomography (CT) of the abdomen and pelvis demonstrated circumferential rectal wall thickening measuring up to 2.3 cm, suspicious for malignancy (Figure 1). Imaging also revealed a large hypodense lesion measuring 8.7 × 10 × 7.5 cm in the posterior right hepatic lobe, highly suspicious for metastatic disease (Figure 2).

Colonoscopy performed the following day revealed a large, friable, ulcerated, nearly circumferential bleeding rectal mass extending into the anal canal. Multiple biopsies were obtained. An ultrasound-guided core needle biopsy of the hepatic lesion was performed concurrently, yielding four 18-gauge core specimens. Histopathologic evaluation demonstrated metastatic adenocarcinoma with extensive dirty necrosis, a finding strongly suggestive of a colorectal primary tumor [6]. Biopsy of the rectal lesion confirmed poorly differentiated rectal adenocarcinoma. Based on imaging findings demonstrating hepatic metastasis and nodal involvement, the malignancy was staged as stage IV colorectal carcinoma (cT3-4, N1-2, M1) according to the American Joint Committee on Cancer staging system [7].

Given the patient’s young age, absence of major comorbidities, and potentially resectable hepatic metastasis, a multidisciplinary tumor board recommended systemic chemotherapy with evaluation for possible staged surgical resection following treatment response. Molecular profiling and microsatellite instability testing were recommended to evaluate for hereditary cancer syndromes and potential targeted therapies. Despite extensive counseling regarding prognosis and treatment options, the patient left the hospital against medical advice prior to initiation of oncologic therapy. After final pathology results confirmed metastatic colorectal adenocarcinoma, the patient was contacted and reported plans to seek care at another institution. At the time of this report, the patient had not yet initiated oncologic treatment.

## Discussion

This case illustrates several hallmark characteristics of EOCRC, including advanced metastatic disease at presentation, distal tumor location, and absence of traditional hereditary risk factors. EOCRC accounts for approximately 10% to 12% of CRC diagnoses and continues to increase globally [2,3]. Younger patients are more likely to present with rectal tumors, advanced-stage disease, and distant metastases, frequently due to delays in diagnosis related to lower clinical suspicion in younger populations [3,4].

Although approximately 20% of CRCs occur in individuals with a positive family history, many EOCRC cases arise in patients without hereditary syndromes [8]. The patient’s long-standing constipation and rectal bleeding were initially nonspecific symptoms that are frequently attributed to benign conditions such as hemorrhoids in younger adults. Studies have demonstrated that younger CRC patients often experience longer symptom-to-diagnosis intervals, which contributes to more advanced disease at the time of diagnosis [4]. High intake of red and processed meats is among the most well-established modifiable risk factors. These dietary patterns are believed to promote carcinogenesis through mechanisms including nitrosamine formation, oxidative stress induced by heme iron, and alterations in the gut microbiome [9]. Western dietary patterns characterized by high fat intake and low fiber consumption may further contribute to chronic intestinal inflammation and metabolic dysregulation. 

From a pathologic perspective, the presence of dirty necrosis was a key diagnostic feature in this case. Dirty necrosis refers to intraluminal accumulation of necrotic cellular debris within malignant glandular structures, reflecting rapid tumor cell turnover. This morphologic feature is strongly associated with colorectal adenocarcinoma and can help distinguish metastatic colorectal tumors from other adenocarcinomas involving the liver [5]. Recognition of this histologic finding is particularly important when evaluating metastatic lesions because it can help determine the primary tumor site and guide oncologic management.

Several features distinguish this case from many previously reported EOCRC cases. These include the absence of identifiable hereditary risk factors, the presence of a large hepatic metastasis at initial presentation, a markedly elevated CEA level, and the presence of extensive dirty necrosis within metastatic liver tissue, which helped confirm the colorectal origin of the tumor. The patient’s markedly elevated CEA level (382 ng/mL) further supported the presence of advanced metastatic disease. Although CEA is not sufficiently specific to serve as a diagnostic test, elevated levels are frequently associated with hepatic metastasis and poorer prognosis in colorectal cancer [10]. Additionally, this case is notable for the relatively young age of the patient at diagnosis (38 years). The increasing incidence of EOCRC has prompted revisions in screening recommendations, with organizations such as the American Cancer Society lowering the recommended screening age from 50 to 45 years [11]. However, this patient presented well below even the revised screening threshold, and the median age typically reported for EOCRC generally occurs in the mid-40s [12,13]. The occurrence of metastatic colorectal carcinoma at this age further underscores the increasingly unpredictable presentation of EOCRC and highlights the need for heightened clinical vigilance when evaluating gastrointestinal symptoms in younger adults.

## Conclusions

This case demonstrates the aggressive presentation of EOCRC in a young adult without identifiable hereditary risk factors. The presence of metastatic disease at diagnosis, combined with distinctive histopathologic features such as dirty necrosis, highlights both the biologic aggressiveness of EOCRC and the diagnostic importance of careful pathologic evaluation.

As the incidence of EOCRC continues to rise globally, clinicians must maintain a high level of suspicion when evaluating rectal bleeding, iron deficiency anemia, or persistent gastrointestinal symptoms in younger adults, even in the absence of classical risk factors. Continued research into the molecular and environmental drivers of EOCRC will be critical to improving early detection and outcomes in this growing patient population.
